# Multicentric Standardization of Protocols for the Diagnosis of Human Mitochondrial Respiratory Chain Defects

**DOI:** 10.3390/antiox11040741

**Published:** 2022-04-08

**Authors:** Nuria Bujan, Constanza Morén, Francesc J. García-García, Alberto Blázquez, Clara Carnicer, Ana Belén Cortés, Cristina González, Ester López-Gallardo, Ester Lozano, Sonia Moliner, Laura Gort, Ester Tobías, Aitor Delmiro, Miguel Ángel Martin, Miguel Ángel Fernández-Moreno, Eduardo Ruiz-Pesini, Elena Garcia-Arumí, Juan Carlos Rodríguez-Aguilera, Glòria Garrabou

**Affiliations:** 1 Inborn Errors of Metabolism Section (IBC U737), Molecular Genetics and Biochemistry Service, Hospital Clínic of Barcelona, IDIBAPS, 08036 Barcelona, Spain; nuribu@gmail.com (N.B.); smoliner@clinic.cat (S.M.); lgort@clinic.cat (L.G.); 2 Muscle Research and Mitochondrial Function Laboratory (U722), Cellex-IDIBAPS, Faculty of Medicine and Health Sciences-University of Barcelona, Internal Medicine Department-Hospital Clínic of Barcelona, 08036 Barcelona, Spain; cmoren1@clinic.cat (C.M.); fjgarcia@clinic.cat (F.J.G.-G.); etobiasb@clinic.cat (E.T.); 3 Mitochondrial Diseases Laboratory (U723), Research Institute, Universitary Hospital 12 de Octubre (Imas12), 28041 Madrid, Spain; alberto.blazquez@salud.madrid.org (A.B.); adelmiro@h12o.es (A.D.); mamcasanueva.imas12@h12o.es (M.Á.M.); 4 Laboratory of Inborn Errors of Metabolism, Biochemistry Service (U701), Laboratoris Clínics, Universitary Hospital Vall d’Hebron, 08035 Barcelona, Spain; ccarnicer@vhebron.net; 5 Bioenergetics and Cell Physiology Service (U729), Central Services of Research, University Pablo de Olavide, 41013 Sevilla, Spain; abcorrod@upo.es; 6 Biochemistry Department (U717), Biomedical Research Institute ‘Alberto Sols’, CSIC, Faculty of Medicine, Autonomous University of Madrid, and Instituto de Investigación Sanitaria Hospital 12 de Octubre (Imas12), 28041 Madrid, Spain; cgparamos@iib.uam.es (C.G.); miguelfm@iib.uam.es (M.Á.F.-M.); 7 Cell and Molecular Biology Department (U727), Veterinary Faculty, University of Zaragoza, 50009 Zaragoza, Spain; esterlop@unizar.es (E.L.-G.); eduruiz@unizar.es (E.R.-P.); 8 Department of Cell Biology, Physiology and Immunology, Faculty of Biology, Universitat de Barcelona (UB), and Institute of Biomedicine of the University of Barcelona (IBUB), 08007 Barcelona, Spain; elozano@ub.edu; 9 Mitochondrial Pathology Laboratory, Research Institute, Universitary Hospital Vall d’Hebron, 08035 Barcelona, Spain; e.garcia@vhebron.net

**Keywords:** mitochondrial respiratory chain, enzyme activity, standardization, diagnosis, mitochondrial disease

## Abstract

The quantification of mitochondrial respiratory chain (MRC) enzymatic activities is essential for diagnosis of a wide range of mitochondrial diseases, ranging from inherited defects to secondary dysfunctions. MRC lesion is frequently linked to extended cell damage through the generation of proton leak or oxidative stress, threatening organ viability and patient health. However, the intrinsic challenge of a methodological setup and the high variability in measuring MRC enzymatic activities represents a major obstacle for comparative analysis amongst institutions. To improve experimental and statistical robustness, seven Spanish centers with extensive experience in mitochondrial research and diagnosis joined to standardize common protocols for spectrophotometric MRC enzymatic measurements using minimum amounts of sample. Herein, we present the detailed protocols, reference ranges, tips and troubleshooting methods for experimental and analytical setups in different sample preparations and tissues that will allow an international standardization of common protocols for the diagnosis of MRC defects. Methodological standardization is a crucial step to obtain comparable reference ranges and international standards for laboratory assays to set the path for further diagnosis and research in the field of mitochondrial diseases.

## 1. Introduction

Mitochondria are responsible for powering cell metabolism, calcium homeostasis and heat production. However, under pathologic conditions, mitochondria are also sources for reactive oxygen species (ROS) and cell death [1,2]. Therefore, impaired mitochondrial function may lead the affected cell, tissue or patient to disease or even death [3,4].

Mitochondrial diseases include both inherited defects and secondary dysfunctions associated with physiologic aging or pathologic conditions, ranging from neurodegenerative to oncologic diseases. They constitute a remarkable diagnostic and research challenge because of their significant heterogeneity in clinical symptoms and the wide range of enzymatic reactions, coenzymes and transporters involved in energy metabolism. Additionally, two different genomes (nuclear and mitochondrial DNA) contain the different genes (approximately 1500 and 37, respectively), all coding for mitochondrial proteins. For every patient or disease, a definite diagnosis requires the identification of the responsible gene associated with a specific biochemical, pathological and clinical profile, which comprises diagnosis that requires the interdisciplinary cooperation of clinicians, pathologists, biochemists and geneticists [5]. In research, the specific identification of the dysfunctional mitochondrial defect is also usually hard to establish. Thus, frequently, the results are not easy to interpret due to tissue-specific genetic or biochemical defects that, in case of MRC, encompass the abnormal organization of MRC complexes and super-complexes, their high molecular size and associated instability.

Therefore, since mitochondrial dysfunction underlies numerous disorders, mitochondrial functional assays are often implemented in clinical and research settings to assess mitochondrial involvement in a physiologic or pathological condition. These assays can be performed in a multitude of biological samples and may include a wide range of parameters such as the quantification of ATP or ROS production [6,7], oxygen consumption [8], membrane polarization [9], oxidative phosphorylation coupling [10] or the assessment of protein folding into mitochondrial supercomplexes by blue native polyacrylamide gel electrophoresis [11], among others. However, in the clinical setting where sensitivity, specificity, precision, reproducibility and linearity are required, the determination of MRC enzyme activities is still the gold standard to assess mitochondrial dysfunction and its contribution to disease.

In humans, MRC is formed by four enzymatic complexes (I-IV) and two mobile electron transporters (coenzyme Q and cytochrome C), driving the electrochemical gradient and proton-motive force for energy production through ATP-proton synthase (complex V). The MRC function can be measured in tissue homogenates, cells or mitochondria-enriched fractions, and the obtained results must be compared to specific reference ranges. MRC analyses are routinely performed in energetic tissues with significant oxidative metabolism, mainly in skeletal muscle, although alternative measurements in less invasive samples such as fibroblasts or peripheral blood mononuclear cells (PBMC) are also measured in clinical settings [12]. Alternatively, the use of mitochondria-enriched fractions requires fresh tissue in significant amounts, and there may be a bias for the positive selection of healthy mitochondria from the global mitochondrial population due to differences in mitochondrial weight [13]. Consequently, in the context of clinical diagnosis, muscle tissue homogenate is the sample of choice for mitochondrial disease evaluation. In research settings, tissue and sample preparation frequently depend on the design, objective and model of study.

Sample collection, tissue homogenization, freezing and storage methods are essential steps for the stability of the mitochondrial enzymes and may vary depending on the centers. Additionally, the spectrophotometric methodologies used for protein quantification and MRC enzyme determinations are very diverse. Differences in several key aspects have been found among different laboratories [14]. In particular, in the experimental conditions used to make the sample accessible to reagents (through detergents solubility, osmotic shock or freezing-and-thawing cycles), the composition and pH of the reaction media, the presence or concentration of substrates and inhibitors, the temperature of measurement and the analytical procedure (specially the time of analysis) are usually different in each laboratory [14]. These methodological differences may hamper result comparisons, the establishment of reference ranges or pathological thresholds and sample reanalysis in other specialized laboratories for a second opinion. Due to these difficulties, several authors have carried out inter-laboratory comparisons [14,15,16], concluding, in all cases, that a strict methodological standardization is necessary to improve the assessment of mitochondrial dysfunction.

In this context, seven Spanish laboratories belonging to the *Centro de Investigación Biomédica en Red de Enfermedades Raras* (CIBERER), working in the diagnostic and research of mitochondrial diseases, started a coordinated program to establish common protocols for MRC enzyme determinations in all laboratories. The present work summarizes this experience and the reference values and adapted protocols to validate and standardize MRC assessment in mitochondrial diagnosis or research, with specific tips for the implementation of these protocols in other laboratories. The methods of this network for the standardization of MRC enzyme measurements were initially based on the protocols of French laboratories [15] to widen the scope of institutions implementing the same methodology, although individual contribution to protocol setup performed by the Italian group of Spinazzi et al., as well as in-house modifications, had been implemented to improve assay performance [16]. In this manuscript, differences in reagents and procedure setup with respect to the previous literature are highlighted, in addition to main troubleshooting assistance. Furthermore, detailed protocols for the implementation of all specific MRC assays are provided within Supporting information in Supplementary Materials.

MRC complex activities were measured spectrophotometrically and were expressed either as absolute (units), specific enzymatic activities (nmol·min^−1^mg of protein^−1^) or normalized to citrate synthase (CS) activity, which is a widely considered biomarker of mitochondrial content [17,18,19]. Herein, we aimed to standardize MRC assays in the seven participant laboratories in which our results would be comparable, combining our experimental experience to overcome methodological pitfalls.

## 2. Materials and Methods

Extensive detailed protocols can be found in Supporting information in Supplementary Materials, and the main differences between proposed methodology and previous French protocols [15] are summarized in Table 1**,** together with troubleshooting solutions for critical methodological steps to improve result robustness.

### 2.1. Reagents

We used the reagents previously recommended for the MRC assay [15], except for decylubiquinol preparation, because this electron donor for complex III assay is extremely sensible to auto-oxidation, and in our hands, the reduction in decylubiquinol with dithionite (previously suggested) led to non-reproducible results. Thus, decylubiquinol preparation was performed by using sodium borohydride [20], which leads to a faster decylubiquinone reduction, requires lower amounts of reagent, prevents the contamination of decylubiquinol with traces of the chemical reducer and extends the stability of the resulting product from 2 h to 3 months (see reagents section in Supporting information).

### 2.2. Procedure

**Figure 1:**
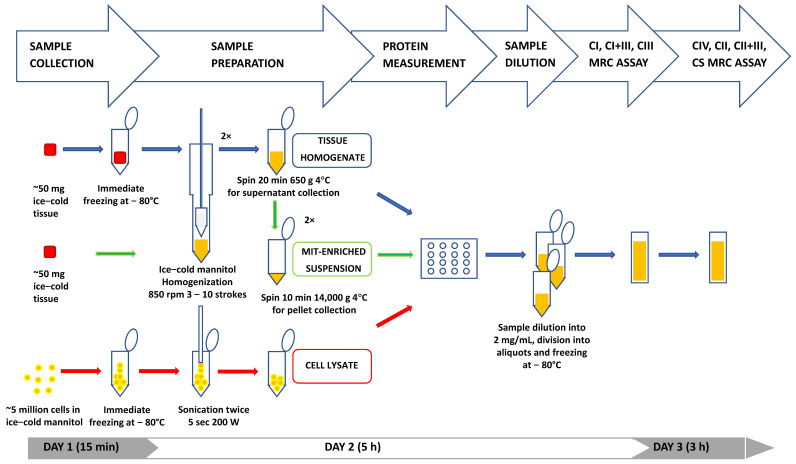
Methodological procedure for sample handling and mitochondrial respiratory chain measurements.

The protocols for MRC measurement were divided into the following: (i) sample collection; (ii) sample preparation (tissue homogenization, cell lysate and mitochondria-enriched preparation); (iii) protein measurement; (iv) MRC enzyme assessments; (v) CS quantification; and (vi) online database setup and result analyses (Figure 1).

As in the Reagents section, only differences with previous procedures have been highlighted [15], although detailed protocols can be found in Supporting information in Supplementary Materials.

(i) Sample collection (expected time: 15 min)

Human sample collection was carried out after written informed consent signature and ethics committee approval were obtained from each institution (C0000128, HCB2017/0808, CEI:18/487, PR(IR)63/2016 and C.I. 2768-N-21). Briefly, muscle, fibroblasts and PBMC samples were obtained for diagnostic purposes following standard medical procedures. These samples were obtained from subjects with clinical suspicion of a potential mitochondrial disease, and the results were used as normality ranges once the mitochondrial defect was ruled out.

Muscle samples were obtained from a biopsy of skeletal muscle (quadriceps, biceps or deltoids) and immediately frozen at −80 ± 5 °C until analysis. The characteristics of the individuals included in this study are summarized in Supplementary Table S1. Samples contaminated with biological fluids such as blood were cleaned by quick paper-tissue absorption (in case of tissue handling) or washed and centrifuged (in the case of cell management). Freezing the sample enables sample transport (from the hospital to the laboratory), sample storage (from collection to analysis) and mitochondrial membrane disruption (enabling substrates to access to the MRC). For muscle samples, a minimum of 50 mg of tissue is required to analyze all MRC activities. Additionally, for each included muscle biopsy, genetic and pathological analyses are performed in parallel to confirm the source of disease and the validity of the sample collected.

For isolated cell measurements (fibroblasts or PBMCs), a collection of a minimum of 5 million cells is recommended. Fibroblasts (collected from a skin punch biopsy and grown in standard culture media) and PBMC (collected in EDTA-plasma tubes and isolated by Ficoll density gradient centrifugation) were washed twice with PBS and frozen at −80 ± 5 °C. For the quantification of MRC in mitochondrial-enriched preparations, fresh tissue or alive cells must be processed before cryopreservation.

(ii) Tissue homogenization, cell lysate and mitochondria-enriched preparation (expected time: 15 min–1 h)

Sample preparation is a critical step to obtain reproducible measurements. Tissue homogenate can be obtained by grinding or by using a polytron device in fresh or frozen tissue.

Frozen muscle samples were quickly thawed on ice-cold mannitol (50 mg of tissue in 200 µL) followed by immediate fragmentation into small pieces using scissors and subsequent homogenization. Although glass–glass homogenizers had been previously recommended [15], in our hands, preparations resulted in suboptimal enzymatic measurements. Consequently, we tested Teflon–glass potters that, in our experience, generated more consistent results. For reproducible results, we recommend keeping tissue and media on ice and handling pestles and mortars with perfect fitting [16]. Homogenization should be performed smoothly, avoiding shearing forces, with the same number of strokes (3–10 strokes), at 850 rpm of speed, alternating fast and slow strokes and using the minimal time needed to obtain homogeneous sample suspensions.

For fibroblasts or PBMC, cells were thawed using 150 µL of ice-cold mannitol media for 5 million cells and then slightly sonicated on ice (twice for 5 s at 200 Watts). Cryopreservation is an optional step from collection to sonication. Both procedures (cryopreservation and sonication) enable the release of MRC complexes to allow proper enzymatic measurements [16].

Mitochondria-enriched fractions from tissues or cells were obtained from fresh biological material (never frozen) through homogenization and sequential centrifugations [13,16,19].

(iii) Protein measurement (expected time: 1 h)

Immediately after sample preparation and before MRC measurements, it is recommended to proceed with protein measurement and subsequent sample dilution to a final concentration of 2 mg/mL in mannitol solution to standardize reaction conditions (sample and solution volumes) and to meet the optimal kinetic requirements for MRC enzyme assays.

Although both Lowry and bicinchoninic acid (BCA) protein assays are frequently used [15], in our hands, the Lowry method was not optimal due to interferences with the mannitol solution. Thus, for precise protein quantification, we used the BCA method run in 96-well microplates by measuring two sample dilution replicates (1/4 and 1/8 duplicates). A set of standards was assessed in parallel together with an internal control sample of known concentration. As a control for BCA measurement, we used a pork muscle homogenate at 2 mg/mL, and it was further used as an internal and inter-lab quality control for MRC assays.

After protein quantification, we recommend preparing aliquots with at least 500 µL of 2 mg/mL tissue homogenate, 200 µL of cell suspension or 100 µL of mitochondria-enriched suspension for further measurements of MRC. Samples should be immediately stored at −80 ± 5 °C in aliquots, if enzymatic assays cannot be performed within the same day to avoid extra freeze–thaw cycles.

(iv) MRC enzyme assessment (expected time: 6 h)

All enzymatic assays were performed using single-wavelength and temperature-controlled spectrophotometers, ideally with multi-cuvette carousel (for simultaneous multi-sample analysis) and controlled software analysis (for standard calculations). Although different temperature settings can be found in the literature [13,14,15,16,17,18], we measured all the MRC activities of human samples at physiological 37 °C. Temperature settings were compared with external calibrated temperature probes. When temperature-controlled devices (water bath and Peltier) showed variations up to 3 °C between nominal and actual temperature in cuvettes, variations were compensated to adjust actual cuvette temperature to 37 °C.

Table 2 summarizes the experimental conditions for MRC assays. Although we recommend performing all assays in one working day (about 6 h), if preferred, they can be performed in two separate days (3 h/day) with comparable results. For instance, we performed sample preparation, protein quantification and dilution into aliquots to a protein concentration of 2 mg/mL in day 1 (2 h procedure). Some aliquots were kept on ice for the subsequent determination of freeze–thaw sensitive MRC activities (day 1), and some aliquots were immediately frozen at −80 ± 5 °C to measure the remaining activities on day 2.

According to inter-laboratory and intra-laboratory results, enzyme assays with higher sensitivity to freeze–thaw cycles that should be performed on day 1 were quantified with complexes I, I + III and III, which showed lower activities in frozen homogenates (data not shown). Consequently, in this multicentric study, less sensitive complexes CII, CII + III, CIV and CS are recommended to be alternatively measured using frozen homogenates on day 2.

Since all MRC enzyme measurements should include an internal control sample run in parallel, all participating centers used frozen aliquots from a common pool of porcine skeletal muscle homogenate at 2 mg/mL, which was also used for protein measurement.

MRC activities were assayed as described previously [15] (see Supporting information in Supplementary Materials), except for complex III and complex IV assays. In these cases, some modifications were introduced to improve both the reproducibility and linearity of these assays. As mentioned in the Reagent section, dithionite was changed to sodium borohydride as the source of electron donor for decylubiquinone reduction into decylubiquinol for complex III measurement [20], and detergent Tween 20 was added to facilitate membrane permeation in the case of complex IV assay. Additionally, a three-fold sample dilution was introduced for both CIII and CIV assays to improve the kinetic conditions [15], while maintaining the buffer concentration constant.

Although activity ranges and assay slopes were analyzed for each individual enzyme assay, we agreed to establish the previously recommended consensus analytic interval of 180 s for all MRC measurements [15], except for CI (60–180 s interval) and CI + III and CIII (0–90 s interval), which improved the reproducibility of the assays. Shorter time intervals are recommended for CIII-related assays to avoid non-linear kinetics due to the exhaustion of redox substrates. In all measurements, when kinetics of the reaction is not linear during the overall time interval (R value ≥ 0.975), the dilution of the sample is mandatory to prevent an underestimation of the potential enzymatic activity.

The Complex V measurement (ATP-proton synthase assay) is not included in most MRC assay protocols [15,16] because of insufficient reliability in frozen tissue or cells, due to negligible mitochondrial ATP hydrolysis in front of overall unspecific cell ATPase activity. Alternatively, this quantification can be performed with polarographic studies in oxygen consumption assays in fresh tissue or alive cells [21]. A spectrophotometric complex V measurement was also possible in mitochondria-enriched suspension from fresh tissue or live cells [22] or, in frozen samples, after the treatment of the cells with percoll and digitonin [23,24,25]. However, in our experience, the reproducibility of the results is usually suboptimal for diagnostic purposes. On the other hand, Blue Native Polyacrylamide gel electrophoresis (BN-PAGE) in combination with in-gel catalytic staining is a powerful tool for the diagnosis of oxidative phosphorylation defects, including complex V deficiency [26].

(v) CS enzyme quantification

The amount of mitochondria present in the samples is estimated by measuring the activity of the enzyme CS [17,18,19]. Although MRC activities can be expressed as specific values referred to as protein content (nmols/min·mg protein), they are usually normalized to CS activity in order to take into account the actual mitochondrial content.

(vi) Database, online work, quality controls and result analysis

The results of MRC enzyme activities of each participating center are systematically collected and updated online in cloud space where protocols, results and batches are recorded and shared among groups. Databases are routinely updated to gather reference control values, which include either the results of center-specific human samples or those of shared pork muscle aliquots used as common inter-group control samples (see Supplementary Table S2). The database was created for a dynamic, numeric, statistic and graphic analysis overview of the recruited results to evaluate center-specific deviations with respect to mean values or center-specific deviations over time (useful to detect batch-derived variations). Additionally, annual calibration samples are routinely analyzed and compared among participating centers to test inter-laboratory variability.

## 3. Results and Discussion

**Figure 2:**
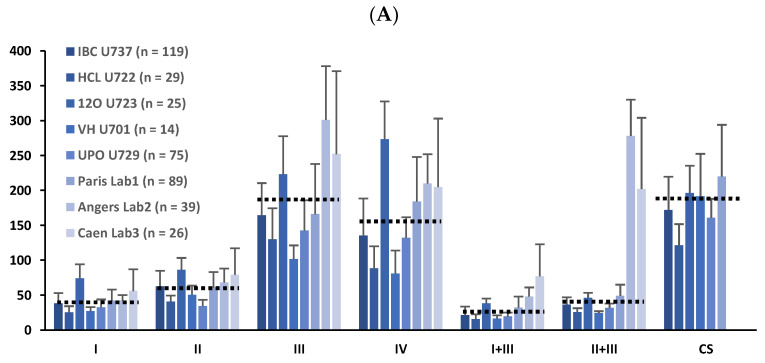

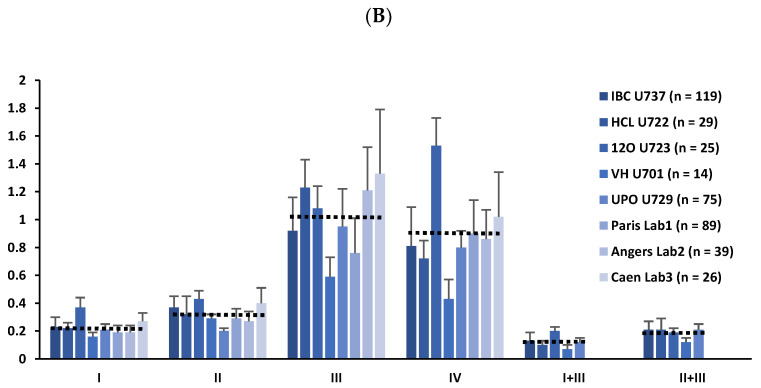
Graphical comparison of data from Table 3 corresponding to mitochondrial respiratory chain enzymatic results of human muscle samples analyzed in five of the seven participating centers (affiliations 1 to 5) and results of three French groups (Medja et al. [15]). Two participating laboratories (affiliations 6 and 7) did not provide data because they focused their activity on animal and cell models. (**A**) Specific MRC enzymatic activities; (**B**) Relative MRC enzymatic activities to CS content.

The present study provides data from seven independent centers summarizing all the experimental steps of MRC standardization using data from human muscle samples. The results of MRC enzymatic activities in human muscle homogenates after protocol standardization are shown in Table 3 and Figure 2, expressed as mean and standard deviation and include specific activities or relative values to CS. Table 4 summarizes the troubleshooting recommendations for different problems potentially arising during protocol set up. Table 5 shows the results of the standardization of MRC assays in other human samples (fibroblasts and PBMC), while internal quality controls can be found in Supplementary Table S2.

The results herein described are comparable to those obtained by the French laboratories [15], including CIII and CIV measurements where we had to introduce some technical modifications for optimal measurements. As shown in Table 3 and Figure 2, specific activities and, in particular, relative enzymatic activities to CS were similar after protocol standardization and achieved reference ranges comparable with the French laboratories. Importantly, inter-laboratory differences became smaller when enzymatic activities were related to CS to normalize MRC relative to mitochondrial extraction efficiency (see coefficient of variation and color distribution of Table 3, Table 5 and Supplementary Table S2). Additionally, the normalization of MRC enzyme activities to the mitochondrial content is a useful tool to correct potential increases in mitochondrial activities that may occur in some mitochondrial diseases to preserve mitochondrial function [1,2,3]. Consequently, in this study, we highly recommend relative MRC assessment to CS in order to minimize inter-laboratory variability [17,18,19].

The protocols herein contained and the methodological troubleshooting shared were the starting point of a diagnostic workflow. Despite this, we do not provide data/examples of specific patient’s diagnosis, as it is the daily practice of all laboratories participating in the present methodological standardization. We use the reference ranges provided in Table 3 and Table 5 for the biochemical characterization of the patient and established a specific diagnosis after cross-checking additional clinical, pathological and genetic information.

The pathological threshold for biochemical mitochondrial disease diagnosis should be set after analyzing the maximal number of control muscle samples available. However, due to invasiveness of muscle biopsies, diagnostic centers usually use those obtained from patients as control samples, when potential pathological mitochondrial conditions have been ruled out. On the other hand, the analysis of previously confirmed pathological samples is also very helpful to validate healthy reference values.

The reference interval is usually established as one or two standard deviations from the average reference value, encompassing the 84th to 95th percentile of the healthy population, in the case of normal distributed parameters. However, alternative statistical calculations are also used (maximum–minimum scores, reference interquartile range, etc.).

Regarding the source of variability for the reference ranges used in the different sites (see Table 3), we did not detect significant demographic differences in age and gender between institutions, except for one site (HCL U722), with an equal parity in males to females’ ratios (as the rest of centers) but a differential age of range onset (starting in adulthood vs. newborn inclusion for the rest of sites). The reason of this center for focusing on reference values extracted from adult individuals is that this site does not diagnose pediatric patients. Interestingly, this institution showed the lowest values of mitochondrial respiratory chain activities (Table 3). Whether aging was or not the causal reason for such differences is still a matter of doubt, since age stratification is not feasible due to reference ranges based on small cohort sample sizes. Remarkably, supporting age and site-specific control characteristics influence on variability, MRC divergence was smaller when the same common control pork sample was evaluated for the same sites (see Supplementary Table S2).

MRC enzyme deficiency may be suspected when enzyme activity is lower than this reference value, particularly if the patient shows clear symptoms of mitochondrial disease and additional evidence of mitochondrial disfunction such as elevated mitochondrial content (CS activity), abnormal anatomopathological findings (either ragged-red fibbers or COX−/SDH+ cells) or genetic variations.

Of note, diagnostic thresholds depend on the source of samples analyzed, as well as reference values and the rate of false positive/negative values assumed. Sample analysis may vary, mainly, depending on size of the cohort, variable distribution (Gaussian or non-parametric) and composition (healthy individuals or both healthy individuals and patients), among others. The rate of sensitivity and specificity assumed (alpha and beta errors) will determine the risk of ascertainment bias in false/positive diagnosis. Thus, each center establishes the pathological threshold based on mean values plus one or two standard deviations (for parametric parameters) or median values and percentiles (for non-parametric parameters), depending on all these considerations [25]. Additionally, in the case of the evaluation of pediatric patients, it is advisable to set range values depending on the patient age due to MRC activity and mitochondrial content drift after the first stages of life [26].

In our experience of multicentric protocol implementation, the network for communication in-person, online, by-phone or electronic mail among the members of all participating centers was essential for the work-in team in terms of troubleshooting and result comparisons. Additionally, the daily update of our online cloud-space databases was useful for the following: (i) record comparable information of control ranges, either from human or reference samples; and (ii) detect site-specific deviations, variations over time and batch-dependent changes. The annual exchange of calibration samples was routinely performed to detect potential bias.

During the setup of MRC measurements, the comparison of our results with previously published data [15] was crucial to optimize our protocols. The aim of this manuscript is to help others with the same purpose.

One important source of variability affecting several MRC assays (complexes I + III, II + III and IV) was the cytochrome c reagent. Further research should be performed to normalize the basal redox balance of this commercial reagent to reduce derived batch-dependent variability.

Despite all the above-mentioned complexities, the standardization of protocols for the validation of MRC measurements is an urgent need for all laboratories studying MRC defects, especially in diagnostic centers. It is difficult to avoid epidemiologic, logistic or sample collection differences between sites, which reflects the complexity that diagnostic and research laboratories face on a daily basis with real world circuits. However, it is possible to minimize the source of experimental variability in the laboratory analyses with standardized and robust experimental protocols.

We strongly recommend setting up the herein reported protocols, as we found them to be reliable and sensitive, and they are specific methods for the study and diagnosis of mitochondrial dysfunction. These protocols are also recommended in the context of research settings, since they provide reliable methodological protocols for the measurement of MRC in humans not only in muscle biopsies but also in cell and animal models, setting the path for international standardization and the comparison of results in experimental networks or the scientific literature. In the case of MRC deficiency, the further association of mitochondrial defects with ROS production, proton leak, ATP decrease, heat imbalance, membrane potential disruption, dysfunctional mitophagy, altered calcium homeostasis or apoptosis may help to establish a link between organelle-to-cell dysfunction and disease.

## 4. Conclusions

1. Mitochondrial respiratory chain enzymatic assays are relevant analytical tools in research and diagnosis with high inter-laboratory variability.
2. Updated and functional standardized protocols, normality ranges and troubleshooting methods are herein provided to overcome the most common experimental challenges.
3. This Spanish multicentric study joins the previous French and Italian initiatives to encourage other countries to establish common international analytical standards.
4. Additional analyses of clinical, genetic and pathological measures should be performed to better understand the contribution of MRC defects to human disease.

## Figures and Tables

**Table 1:** Main differences between our multicentric study and the French network’s study (Medja et al. [15]) and critical steps of protocol standardization.

Difference	French Network		Spanish Network	Aim
Homogenization	Glass-glass potters		Teflon-glass potters	Avoiding MRC damage
Protein quantification	BCA or Lowry		BCA	Avoiding Mannitol interference in protein quantification
Time for MRC assays	One day		Feasible in one day but divided into two days (or as much as MRC assays) by using aliquots	Adapting protocols into laboratory standards
MRC Complex III measurement	Decylubiquinol reduction through dithionite		Decylubiquinol reduction through sodium borohydride	Longer and more stable reagent reduction
MRC Complex III measurement	No detergent into the reaction mix		Tween 20	Linear kinetic ranges
MRC Complex III measurement	No sample dilution		3-fold sample dilution	Linear kinetic ranges
MRC Complex IV measurement	No sample dilution		3-fold sample dilution	Linear kinetic ranges
MRC analysis	No specific analysis time interval		Standardized and specific analysis time intervals for each assay	Result consistency
Common control sample	Site-specific		Common to all centers	Allowing inter-site result comparison and site-specific problem detection
Online batch reagent register	Non specified		Batch reagent recorded and compared among groups	Allowing inter-site result comparison and batch-specific problem detection
**Critical Steps**		**Recommendation**		
Sample Collection		It is critical to minimize time from sample collection to cryopreservation and fast ice-cold thawing before homogenization		
Sample Preparation		Homogenization of tissue in fragments weighing at least 50 mg; otherwise, sample fractions are lost through the homogenization process		
		Extensive training in homogenization to obtain reliable measurements		
Protein quantification and sample dilution		Protein measurement allowed sample dilution to 2 mg/mL in mannitol prior to MRC measurements to meet the same kinetic requirements		
MRC enzyme measurement		Temperature stability maintained at 37 °C (human physiologic conditions)		
		Introducing control samples in protein quantification and MRC assays		
		When possible, analysis of patients and control samples in parallel		
		Online network: cloud databases to register reference ranges of human samples, control samples and reagent traceability		

**Table 2:** Standardized experimental conditions for mitochondrial respiratory chain assays.

	CI	CII	CIII	CIV	CI + III	CII + III	CS
**Wavelength (nm)**	340	600	550	550	550	550	412
**Buffer**	50 mM KP pH7.5	25 mM KP pH 7.5	25 mM KP pH 7.5	50 mM KP pH 7.0	50 mM KP pH 7.5	20 mM KP pH 7.5	100 mM Tris-HCl pH 8.1
**Substrates**	100 µM NADH 100 µM DQ	20 mM Succinate 50 µM DCPIP 100µM DQ	75 µM Cytochrome C 100 µM DQH2	100 µM Reduced Cytochrome C	200 µM NADH 100 µM Cytochrome C	20 mM Succinate 100 µM Cytochrome C	300 µM Acetyl-CoA 100 µM DTNB 500 µM Oxaloacetate
**Other reagents**	BSA 3.75 mg/mL	BSA 2 mg/mL	0.1 mM EDTA 0.025% Tween 20 *v*/*v* 0.5 mM KCN		BSA 1 mg/mL 1 mM KCN	BSA 2 mg/mL	0.1% Triton X-100
**Specific inhibitor**	12.5 µM Rotenone	1 mM KCN	Aa 10 µg/ml		12.5 µM Rotenone	1 mM KCN	
**Muscle homogenate**	40 µg protein	40 µg protein	3-fold diluted 20 µg protein	3-fold diluted 40 µg protein	40 µg protein	40 µg protein	40 µg protein
**Preincubation time**	5 min	5 min	5 min	5 min	5 min	5 min	5 min
**Baseline reaction**	-	3 min	-	-	-	Control without sample	4 min
**Initiator of the reaction**	100 µM NADH	100 µM DQ	100 µM DQH2	Sample	200 µM NADH	100 µM Cytochrome C	500 µM Oxaloacetate
**Total assay time(*selected interval*)**	3 min (*consider 1–3*)	3 min (*consider 0–3*)	3 min (*consider 0–1.5*)	3 min (*consider 0–3*)	3 min (*consider 0–1.5*)	3 min (*consider 0–3*)	4 min (*consider 0–3*)
**ε (mmol^−1^ cm^−1^)**	6.2	19.2	18.5	18.5	18.5	18.5	13.6
**Calculation factor**	4032.3	1302.1	8108.1	4054.2	1351.4	1351.4	1838.2

The corresponding enzyme activity (nmol·min^−1^·mg of protein^−1^) is as follows: (∆absorbance/min) × (Calculation factor indicated in Table 2). Aa, Antimycin A; Ac-CoA, Acetyl coenzyme A; ATP, Adenosine triphosphate; BCA, bicinchoninic acid; BSA, Bovine Serum Albumin; CI, complex I or NADH:ubiquinone oxidoreductase; CII, complex II or succinate:ubiquinone oxidoreductase; CIII, complex III or coenzyme Q:cytochrome c oxidoreductase; CIV, complex IV or cytochrome c oxidase; CV, complex V or ATPase; CI + II, complex I + II or NADH:cytochrome c oxidoreductase; CII + III, complex II + III or succinate:ubiquinone-ubiquinol:cytochrome c oxidoreductase; COX, cytochrome c oxidase; CS, citrate synthase activity, DCPIP, 2,6-Dichloroindophenol sodium salt hydrate; DQ, Decylubiquinone; DQH2, Decylubiquinol; DTNB, 5,5′-Dithiobis(2-nitrobenzoic acid); EDTA, Ethylenediaminetetraacetic acid disodium salt solution; EtOH, ethanol; HCl, Hydrogen chloride; KCN, Potassium cyanide; KP, Potassium phosphate buffer; NADH, β-Nicotinamide adenine dinucleotide; MB, mannitol buffer; MRC, Mitochondrial respiratory chain; PBMC, peripheral blood mononuclear cells; ROS, reactive oxygen species; OXS, oxidized solution; RS, reduced solution; SDH, succinate dehydrogenase; UCS, units of CS; β-Nicotinamide adenine dinucleotide; µg: Micrograms; min: Minute ε: Molar absorbance coefficient.

**Table 3:** Mitochondrial respiratory chain enzymatic results of human muscle samples analyzed in five of the seven participating centers (affiliations 1 to 5) and comparison with respect to reports of three French groups (Medja et al. [15]). Two participating laboratories (affiliations 6 and 7) did not provide data because they focused their activity on animal and cell models.

Complex	Activity	IBC U737		HCL U722		12O U723		VH U701		UPO U729		Paris Lab ^1^		Angers Lab ^2^		Caen Lab ^3^	
		(n = 119)		(n = 29)		(n = 25)		(n = 14)		(n = 75)		(n = 89)		(n = 39)		(n = 26)	
		Mean ± SD	CV	Mean ± SD	CV	Mean ± SD	CV	Mean ± SD	CV	Mean ± SD	CV	Mean ± SD	CV	Mean ± SD	CV	Mean ± SD	CV
**I**	Specific	37.99 ± 14.86	39.1	25.09 ± 9.09	36.2	74.12 ± 19.95	26.9	27.20 ± 5.82	21.4	32.52 ± 11.33	34.8	42 ± 16	38.1	42 ± 8	19.0	56 ± 31	55.4
	Relative to CS	0.23 ± 0.07	30.4	0.22 ± 0.04	18.2	0.37 ± 0.07	18.9	0.16 ± 0.03	18.8	0.21 ± 0.04	19.0	0.19 ± 0.05	26.3	0.19 ± 0.05	26.3	0.27 ± 0.06	22.2
**II**	Specific	62.46 ± 22.54	36.1	40.60 ± 8.78	21.6	86.03 ± 17.34	20.2	50.37 ± 13.41	26.6	34.32 ± 9.03	26.3	61 ± 22	36.1	68 ± 20	29.4	79 ± 38	48.1
	Relative to CS	0.37 ± 0.08	21.6	0.32 ± 0.13	40.6	0.43 ± 0.06	14.0	0.29 ± 0.03	10.3	0.20 ± 0.02	10.0	0.29 ± 0.07	24.1	0.27 ± 0.07	25.9	0.40 ± 0.11	27.5
**III**	Specific	164.21 ± 46.29	28.2	129.77 ± 44.82	34.5	222.94 ± 54.77	24.6	101.44 ± 19.82	19.5	142.45 ± 43.90	30.8	166 ± 72	43.4	301 ± 77	25.6	252 ± 119	47.2
	Relative to CS	0.92 ± 0.24	26.1	1.23 ± 0.20	16.3	1.08 ± 0.16	14.8	0.59 ± 0.14	23.7	0.95 ± 0.27	28.4	0.76 ± 0.25	32.9	1.21 ± 0.31	25.6	1.33 ± 0.46	34.6
**IV**	Specific	135.15 ± 53.34	39.5	88.47 ± 31.63	35.8	273.31 ± 54.10	19.8	80.87 ± 33.15	41.0	132.09 ± 29.47	22.3	184 ± 64	34.8	210 ± 42	20.0	205 ± 98	47.8
	Relative to CS	0.81 ± 0.28	34.6	0.72 ± 0.13	18.1	1.53 ± 0.20	13.1	0.43 ± 0.14	32.6	0.80 ± 0.12	15.0	0.90 ± 0.24	26.6	0.86 ± 0.21	24.4	1.02 ± 0.32	31.4
**I + III**	Specific	21.41 ± 12.20	57.0	15.61 ± 6.92	44.3	38.17 ± 6.80	17.8	16.22 ± 4.88	30.1	19.36 ± 5.62	29.0	32 ± 16	50.0	NA	NA	NA	NA
	Relative to CS	0.13 ± 0.06	46.1	0.10 ± 0.03	30.0	0.20 ± 0.03	15.0	0.07 ± 0.03	42.9	0.12 ± 0.03	25.0	NA	NA	NA	NA	NA	NA
**II + III**	Specific	36.33 ± 10.57	29.1	25.73 ± 5.53	21.5	46.03 ± 7.35	16.0	24.21 ± 2.75	11.4	31.68 ± 6.18	19.5	49 ± 16	32.7	48 ± 13	27.1	77 ± 46	59.7
	Relative to CS	0.21 ± 0.06	28.6	0.21 ± 0.08	38.1	0.19 ± 0.03	15.8	0.12 ± 0.03	25.0	0.21 ± 0.04	19.0	NA	NA	NA	NA	NA	NA
**CS**	Specific	172.02 ± 47.6	27.7	121.48 ± 30.23	24.9	196.19 ± 39.28	20.0	191.55 ± 60.88	31.8	160.87 ± 27.02	16.8	220 ± 74	33.6	278 ± 52	18.7	202 ± 102	50.5

Results are expressed as mean ± 1 standard deviation in nmols·min^−1^·mg of protein^−1^ and Coefficient of Variation (CV). Participating centers: IBC, HCL, 12O, VH and UPO (filiations 1–5). French laboratories (Medja et al., 2009 [15]): 1. La Salpêtrière hospital (Paris, France), 2. CHU d’Angers (Angers, France) and 3. CHU de Caen (Caen, France); I–IV: Enzymatic activities of MRC complexes I to IV; CS: Enzymatic activity of citrate synthase.

**Table 4:** Troubleshooting recommendations.

Problem	Cause	Solution
Low MRC enzymatic activities	Excessive homogenization deteriorates sample stability	Normalize by citrate synthase and, if possible, repeat homogenization
	Incorrect protein quantification with overestimation of real value	Normalize by citrate synthase and, if possible, repeat protein quantification
	Temperature of spectrophotometer is below 37 °C	Check temperature of the spectrophotometer
	Different wavelength than required for the assay	Check wavelength requirement of the specific assay
	Reaction medium or required reagent for the assay has been deteriorated	Check reagent storage: protection from light, cold storage, too long time since preparation
	Not enough sample has been added to the assay	Duplicate or triplicate sample amount
	Excessive sample has been added to the assay (usually reaction will not be linear)	Decrease by half or third sample amount
Low Complex I activity	NADH has been oxidized	Check macroscopic appearance of stored NADH (NADH should be dry and white)
	Inhibition is not efficient	Check proper Rotenone addition and mixture into the cuvette (appropriate handling and pipetting)
Low Complex II activity	Lack of electron acceptor (reaction product)	Check DCPIP expiration date
Low Complex III activity	Lack of electron donor (substrate product)	Check decylubiquinol expiration date
	Inhibition is not efficient	Check proper antimycin a addition into cuvette (appropriate handling and pipetting)
Low Complex IV activity	Lack of electron donator (substrate product)	Has been reduced cytochrome c extemporaneously prepared?
		Check if absorbance of reduced cytochrome c is still in the proper range (90–95% oxidized one)
		Change of cytochrome c batch
Low Complex I + III activity	Inhibition is not efficient	Check proper rotenone addition into cuvette (appropriate handling and pipetting)
Low citrate synthase	Reaction medium or required reagent for the assay has been deteriorated	Check acetyl coenzyme a, oxaloacetate and DTNB integrity
High MRC enzymatic activities	Incorrect protein quantification (below real value) increased calculations	Normalize by CS and, if possible, repeat protein quantification

DCPIP: 2,6-Dichloroindophenol sodium salt hydrate; DTNB: 5,5′-Dithiobis (2-nitrobenzoic acid); NADH: β-Nicotinamide adenine dinucleotide; Complexes I-IV: Enzymatic activities of MRC complexes I to IV; CS: Enzymatic activity of citrate synthase; MRC: mitochondrial respiratory chain.

**Table 5:** Mitochondrial respiratory chain enzymatic results of control human fibroblasts and peripheral blood mononuclear cells (PBMC) in two participating centers compared to previous reports by French network [15].

		Fibroblasts				PBMC	
		IBC U737		French Group		HCL U722	
		(n = 28)		(n = 50)		(n = 11)	
Complex	Activity	Mean ± SD	CV	Mean ± SD	CV	Mean ± SD	CV
**I**	Specific	NA	NA	NA	NA	28.99 ± 8.36	28.8
	Relative to CS	NA	NA	NA	NA	1.18 ± 0.34	28.8
**II**	Specific	29 ± 6	20.7	27 ± 8	29.6	19.42 ± 1.81	9.3
	Relative to CS	0.64 ± 0.11	17.1	0.36 ± 0.10	27.8	0.85 ± 0.14	16.5
**III**	Specific	36 ± 12	33.3	55 ± 17	30.9	28.04 ± 4.31	15.4
	Relative to CS	0.78 ± 0.28	35.9	0.79 ± 0.25	31.6	1.37 ± 0.20	14.6
**IV**	Specific	45 ± 12	26.6	83 ± 15	18.1	16.44 ± 3.04	18.5
	Relative to CS	1.00 ± 0.15	15	1.12 ± 0.22	19.6	0.63 ± 0.11	17.5
**I + III**	Specific	28 ± 14	50	24 ± 7	29.2	NA	NA
	Relative to CS	0.55 ± 0.17	30.9	NA	NA	NA	NA
**II + III**	Specific	16 ± 4	25	30 ± 7	23.3	NA	NA
	Relative to CS	0.33 ± 0.1	30.3	NA	NA	NA	NA
**CS**	Specific	51 ± 11	21.6	79 ± 18	22.8	24.30 ± 7.61	31.3

Results are expressed as mean ± 1 standard deviation in nmol·min^−1^·mg protein^−1^ and Coefficient of Variation (CV). IBC (U737 CIBERER): Sección de Errores Congénitos del Metabolismo (IBC), Servicio de Bioquímica y Genética Molecular, Hospital Clinic de Barcelona, IDIBAPS and CSIC, Barcelona, Spain; HCL (U722 CIBERER): Laboratorio de Investigación Muscular y Función Mitocondrial, Cellex-IDIBAPS, Facultad de Medicina-Universidad de Barcelona, Departamento de Medicina Interna-Hospital Clínic de Barcelona, Barcelona, Spain; All of them from the Centro de Investigación Biomédica en Red (CIBER), Sección de Enfermedades Raras (CIBERER); French laboratory: La Salpêtrière hospital (Paris, France); I-IV: Enzymatic activities of MRC complexes I to IV; CS: Enzymatic activity of citrate synthase; NA: Not available.

## Data Availability

All data used to support the findings of this study are included within the article.

## Supplementary Materials

The following supporting information can be downloaded at: https://www.mdpi.com/article/10.3390/antiox11040741/s1, Supplementary File: Supporting information; Table S1: Characteristics of the samples and individuals from the different sites of the study included as reference ranges (Table 3 and Table 5); Table S2: Representative data of the control porcine sample.
