# Supplementary material for: Multicentric Standardization of Protocols for the Diagnosis of Human Mitochondrial Respiratory Chain Defects
Source: Antioxidants (Basel). 2022 Apr 8;11(4):741. doi: 10.3390/antiox11040741 (PMC9027926; doi:10.3390/antiox11040741)
Supplement: Supplementary file 1 [file antioxidants-11-00741-s001.zip › antioxidants-1603519-supplementary information.pdf]

SUPPLEMENTARY INFORMATION

1. Reagents **2**

2. Reagent setup **3**

3. Procedure **7**

(i) ***Sample collection*** **7**

(ii) ***Sample preparation*** **7**

Tissue Homogenization **7**

Cell lysate (fibroblasts or PBMC) **8**

Mitochondria-enriched preparation **8**

(iii) ***Protein measurement*** **9**

(iv) ***MRC enzyme assessment*** **9**

- **COMPLEX I (NADH:ubiquinone oxidoreductase, CI)** **9**
- **COMPLEX II (succinate:ubiquinone oxidoreductase, CII)** **11**
- **COMPLEX I + III (NADH:cytochrome c oxidoreductase, CI+III)** **12**
- **COMPLEX II+III (succinate:ubiquinone-ubiquinol:cytochrome c oxidoreductase, CII+III)** **13**
- **COMPLEX III (decylubiquinol: cytochrome C oxidoreductase, CIII)** **14**
- **COMPLEX IV (Cytochrome C oxidase, CIV)** **15**

(v) ***Citrate Synthase (CS) enzyme quantification*** **16**

1. REAGENTS

Acetyl coenzyme A lithium salt, ≥93% (HPLC) (AcCoA; Sigma, cat. no. A2181). Store at -20°C.
Albumin from bovine serum, lyophilized powder, essentially fatty acid free, ≥96% (agarose gel electrophoresis) (BSA; Sigma, cat. no. A6003). Store at 0-6°C.
Antimycin A from Streptomyces sp. (Sigma, cat. no. A8674). Store at -20°C. CAUTION! Highly toxic product.
Cytochrome c from equine heart (Sigma, cat. no. C7752). Store at -20°C. Keep protected from light. CRITICAL, not all the lots work similarly. Evaluation of the lot has to be performed before using it.
Decylubiquinone ≥97% (HPLC) 25mg (DB; Sigma, cat. no. D7911 or Enzo, cat. no. BM1-CM115-0010) Store at -20°C. CRITICAL, not all the lots work similarly. Evaluation of the lot has to be performed before using it.
2,6-Dichloroindophenol sodium salt hydrate, suitable for vitamin C determination, BioReagent (DCPIP; Sigma, cat. no. D1878). Keep container tightly closed in a dry and well-ventilated place.
Dipotassium hydrogen phosphate (Potassium phosphate dibasic; Prolabo, cat. no. 33612.268)
5, 5′-Dithiobis(2-nitrobenzoic acid), ≥98% BioReagent, suitable for determination of sulfhydryl groups (DTNB; Sigma, cat. no. D8130). Keep container tightly closed in a dry and well-ventilated place. CAUTION! Irritant product.
Dimethyl sulfoxide (DMSO; Sigma, cat. no. D2650). Keep container tightly closed in a dry and well-ventilated place.
Ethylenediaminetetraacetic acid disodium salt solution, for molecular biology, 0.5 M in H_2O, DNase, RNase, NICKase and protease, none detected (EDTA; Sigma, cat. no. E7889). Keep container tightly closed in a dry and well-ventilated place.
D-Mannitol, BioXtra, ≥98% (GC) (Sigma, cat. no. M9546). Keep container tightly closed in a dry and well-ventilated place.
β-Nicotinamide adenine dinucleotide, reduced disodium salt hydrate (NADH; Sigma, cat. no. N8129). Keep the powder dry and store at -20°C.
Oxaloacetic acid, ≥97% (Sigma, cat. no. O4126). Store at -20°C.
Potassium cyanide, puriss. p.a., ACS reagent, reag. Ph. Eur., ≥97.0% (KCN; Sigma, cat. no. 31252). Keep container tightly closed in a dry and well-ventilated place. CAUTION! Highly toxic product.
Potassium hexacyanoferrate (III), ACS reagent, ≥99.0% (Sigma, cat. no. 244023). Keep container tightly closed in a dry and well-ventilated place.

Potassium dihydrogen phosphate (Potassium phosphate monobasic; Calbiochem, cat. no. 529568).
Rotenone, ≥95% (Sigma, cat. no. R8875). Keep container tightly closed in a dry and well-ventilated place. CAUTION! Highly toxic product.
Sodium borohydride, powder, ≥ 98.0% ($NaBH_4$; Sigma, cat. no. 452882). Keep container tightly closed in a dry and well-ventilated place. CAUTION! Highly toxic product.
Sodium hydrosulfite technical grade, 85 % (Dithionite; Sigma, cat. no. 157953). Keep container tightly closed in a dry and well-ventilated place. CAUTION! Highly toxic product.
Succinic acid, ReagentPlus®, ≥ 99.0 % (Sigma, cat. no. S7501). Keep container tightly closed in a dry and well-ventilated place.
Sucrose, ≥ 99.5 % (GC) (Sigma, cat. no. S9378). Keep container tightly closed in a dry and well-ventilated place.
Tris-hydroxymethylaminomethane (TRIS, Trometamol; Prolabo, cat.no 28811.295). CAUTION! Irritant product.
Triton™ X-100, BioUltra, for molecular biology (Sigma, cat. no. 93443). CAUTION! Irritant product.
Tween 20, viscous liquid (Sigma, cat. no. P1379). Keep container tightly closed in a dry and well-ventilated place.

2. REAGENT SETUP

Acetyl coenzyme A (AcCoA) (10 mM): Weigh AcCoA and dilute at 0.112 ml/mg in distilled water. Store in 150 µL aliquots at -20 ° C.
Antimycin A (2.5 mg/ml): Weigh Antimycin A and dilute at 0.4 ml/mg in 95% ethanol. Store in 30 µL aliquots at -20 ° C. Keep protected from light.
Bovine serum albumin (BSA) (50 mg/ml): Weigh BSA and dilute at 20 µL/mg of distilled water. Store in 1 ml aliquots at -20 °C. Aliquots can be thawed twice.
Cytochrome C (1 mM). Weigh cytochrome c and dilute at 80.8 µL/mg in distilled water. Keep protected from light. CRITICAL Extemporaneous preparation reagent
Cytochrome C reduced solution (100 µM, pH 7.0): Weigh cytochrome c and dilute at 0,808 ml/mg in 50 mM KP at pH 7.0. Keep protected from light. CRITICAL Extemporaneous preparation reagent. See preparation protocol of 1 mM reduced cytochrome c.

Decylubiquinone (DB) (25 mM): Dissolve the content of 25 mg DB vial in 1.5 ml of DMSO to obtain a 50 mM solution. Dilute this solution 1/2 with DMSO to obtain a 25 mM solution. Store in aliquots at -20 ° C.
DB (40 mM): Dissolve the content of 25 mg DB vial in 1938 µL of 100% EtOH. Keep vial tightly closed, and protected from light. Store at -20 ° C.
2,6-Dichloroindophenol (DCPIP) (5 mM): Dissolve 72.5 mg of DCPIP in 50 ml of distilled water. Store at 0-6°C. The solution is stable up to one month at 4 ° C.
Decylubiquinol (DBH2). See preparation protocol of 10 mM decylubiquinol solution.
5, 5′-Dithiobis (2-nitrobenzoic acid) (DTNB) (15 mM): Weigh DNTB and dilute at 0.168 ml/mg in 95% EtOH. Store in 100 µL aliquots at -20 °C. Keep protected from light.
DTNB (5 mM): Dilute 1/3 from stock 15 mM in 95% EtOH just before use. Store in 100 µL aliquots protected from light.
Potassium cyanide (KCN) (10 mM): Dissolve 1mg of KCN and dilute at 1.536 ml/mg in distilled water. CRITICAL Extemporaneous preparation reagent.
Mannitol buffer: Dissolve 2.05 g of mannitol, 1.28 g of sucrose and 60.6 mg of TRIS in 40 ml of distilled water. Add 10 µL of 0.5 M EDTA. Adjust pH to 7.2 with HCl and adjust the volume to 50 ml.
Nicotinamide adenine dinucleotide (NADH) (2mM): Weigh NADH and dilute at 0.685 ml/mg in distilled water. CRITICAL Extemporaneous preparation reagent.
Oxaloacetic acid (10 mM): Weigh oxaloacetic acid and dilute at 0.758 ml/mg in 0.1 M TRIS at pH 8.1. CRITICAL Extemporaneous preparation reagent.
Potassium phosphate buffer (50 mM KP, pH 7.0). Titrate 50 mM Potassium phosphate dibasic with 50 mM Potassium phosphate monobasic up to a pH of 7.0. Store in 50 ml aliquots at 0-6 °C. The solution is stable up to 2-3 months at 4 °C. For long-term storage, store aliquots at -20 ° C.
Potassium phosphate buffer (500 mM KP, pH 7.5). Titrate 500 mM Potassium phosphate dibasic with 500 mM Potassium phosphate monobasic up to a pH of 7.5. Store in 50 ml aliquots at 0-6 °C. The solution is stable up to 2-3 months at 4 °C. For long-term storage, store aliquots at -20 ° C.
Rotenone (2.5 mM): Weigh rotenone and dilute at 1.014 ml/mg in a 1:1 mixture of 95% EtOH/ DMSO. Store in 50 µL aliquots at -20 ° C. Keep protected from light.
Succinic acid (200 mM) Dissolve 472,4 mg of succinic acid in 10 ml of distilled water. Adjust pH to 7.4 with KOH. Adjust the volume to 20 ml. Store in 500 µL aliquots at -20 °C.

TRIS (1 M, pH 8.1). Dissolve 6.06 g of TRIS in 30 ml of distilled water. Adjust pH to 8.1 with HCl. Adjust the volume to 50 ml. Store at 0-6 °C. The solution is stable up to 2-3 months at 4 °C. For long-term storage, store aliquots at -20 ° C.
TRIS (100 mM, pH 8.1). Dilute 1M TRIS solution to 100mM with distilled water. Store at 0-6 °C. The solution is stable up to 2-3 months at 4 °C. For long-term storage, store aliquots at -20 ° C.
Triton™ X-100, 10 % in H2O. Dissolve 100 µL of Triton X-100 to 1 ml with distilled water, store at 4 °C.
Tween 20, 2.5 % v/v. Dissolve 25 µL of Tween 20 to 1 ml with distilled water, store at 4 °C.

Preparation of 1 mM reduced cytochrome c solution:

- Initial solution: In a 15 ml tube, prepare 12 to 13 ml of 100 µM cytochrome c in 50 mM KP, pH 7 (see reagent setup).

- Total oxidized 100 % solution (SOX): Add few grains of potassium ferricyanide to 1 ml of initial solution cytochrome c (the colour of the solution turns red- dark brown).

- Total reduced 100 % solution (SRT): Add few grains of sodium dithionite to 2 ml of initial solution cytochrome c (the colour of the solution turns pink salmon).

Make a blank with air and read the SOX solution at 550 nm. The absorbance should be about 0.75 for a 1 mM solution. Use SOX solution as a blank and read the SRT solution at 550 nm. This value is considered as 100% reduction.

- Titration of the initial solution with SRT
Transfer an aliquot (50 or 100 µL) of the solution SRT in the initial solution and read the absorbance. Continue adding volumes to achieve an absorbance between 90 % and 95 % of the absorbance of the SRT solution.

ATTENTION: the increment of absorbance may be faster or slower depending on the excess of dithionite in SRT solution.

CRITICAL Extemporaneous preparation reagent

Preparation of 10 mM decilubiquinol (DBH2) solution:

1. Add 10 µmol of decylubiquinone (DB) (200 µL of 40 mM DB, see reagent setup) to a glass tube.
2. Add 1 ml of 100% Ethanol.
3. Add 1 ml of distilled water.
4. Add exactly 12 mg of BH_4Na to the glass tube. Vortex and allow the mixture to settle. Repeat vortexing until the solution becomes colourless.

5. Add 2 drops of concentrated HCl and check that the reducing agent has been completely dissolved and the solution has become transparent.

6. Add 1 ml of diethyl ether and vortex the mixture thoroughly. Add 1 ml more of diethyl ether and vortex again.

7. Add 1 ml of hexane and vortex the mixture thoroughly. Let stand to allow phase separation.

8. Recover the upper organic phase into another glass tube. CAUTION Do not to take the lower phase.

9. Add 1 ml of 2 M NaCl and vortex. Let stand to allow phase separation and collect the upper organic phase into another glass tube. CAUTION Avoid contamination with the lower phase.

10. Dry upper organic phase in nitrogen atmosphere.

11. Add 300 µL of 100% Ethanol and vortex the mixture thoroughly.

12. Make a spectral scan, in the range of 200-400 nm, of the obtained solution in a quartz cuvette, to verify that the absorbance peak is located at 289 nm. Add to the cuvette 12.5 µL of DBH2 and 987.5 µL of Ethanol. Read at 289 nm.

13. Calculate DBH2 concentration (Ci) (∑mM, 289nm DBH2 = 4 $mM^{-1}·cm^{-1}$, dilution factor = 1000 / 12.5 = 80): Ci = (Abs289nm / 4) x 80.
To obtain a 10 mM solution of DBH2, add to the solution an equal volume Vf - 300 µL

14. Control checking: Absorbance at 289 nm of the diluted 10 mM DBH2 (12.5 µL of 10 mM DBH2 and 987.5 µL of EtOH) should be around 1 UA.

15. Store in aliquots at -80±5 °C (stability: 2-3 months). CRITICAL Add a drop of concentrated HCl before dividing into aliquots. Use dark Eppendorf tubes previously filled with a nitrogen stream (anaerobic atmosphere).

3. PROCEDURE

(i) *Sample collection*

- TISSUE: Muscle must be quickly frozen and stored at -80±5 ° C or in liquid nitrogen after cleaning potential contamination by biological fluids of the tissue through fast paper-tissue absorption.

- CELLS (FIBROBLASTS OR PBMC): Remove potential haemoglobin or phenol red interference by washing at least 5 million cells twice in cold PBS (5-10 minute centrifugation 400-1,000 g) and remove supernatant. Re-suspend the pellet in ice-cold mannitol buffer (MB) (approximately 150 µL per 5 million cells) and store immediately frozen at -80±5°C unless sonication has to be performed. Sonication can be optionally performed before or after freezing.

- MITOCHONDRIA-ENRICHED SUSPENSION: Collect tissue or cell sample in ice-cold MB and proceed immediately to mitochondrial isolation avoiding freezing steps.

(ii) *Sample preparation*

- **TISSUE HOMOGENIZATION:**

All steps involved in the preparation of tissue, the homogenization and the preparation of aliquots must be performed on ice.

1. Pre-cool the glass Potter homogenizer on ice before starting homogenization.

2. Take a frozen piece of 40-50 mg of tissue and cut into fragments without thawing.

3. Add 200 µL of cold MB in the Potter, and then add the tissue fragment.

4. Homogenize at 0-6 ° C with a Teflon plunger motor-driven, three to ten controlled up-down strokes at 800-900 rpm are often enough. Stop when the solution is homogeneous. Avoid over-homogenization and consequent heating of the solution.

5. Clean the plunger with 100 µL of cold MB.

6. Transfer the homogenate into a 1.5 ml microtube with a Pasteur pipette and centrifuge 20 minutes at 650 g at 0-4 ° C.

7. Transfer the supernatant into a microtube.

8. Re-suspend the pellet with 200 µL MB, transfer the solution to the previously used Potter and repeat steps 4, 5 and 6. In this case, add 100 µl MB to clean the glass Potter, instead of the plunger.

9. Pool the supernatant (free of nucleus) of the second centrifugation with the first one.

10. Keep the supernatant (homogenate) on ice at 0-6 ° C and measure the total protein concentration.

11. Dilute supernatant (homogenate) to 2 mg/ml. 500 µl is the volume recommended.

12. Respiratory chain complexes can be analysed within the same day in the supernatant (homogenate). Alternatively, it should be rapidly stored at -80±5 ° C or in liquid nitrogen, to determine the various complexes either in other day or distributed in two or more days after homogenization (preferably consecutive days). It is recommended to divide the homogenate into aliquots to be thawed before the enzymatic assay.

- **CELL LYSATE (fibroblasts or PBMC)**

1. In case that sample has been frozen, thaw on ice for sonication. Recommended conditions are: double sonication during 5 seconds at 200 Watts.

2. Keep on ice at 0-6 ° C and measure the total protein concentration.

3. Dilute cell lysate to 2 mg/mL. 200 µl is the volume recommended.

4. Respiratory chain complexes can be analysed within the same day in the cell lysate. Alternatively, it should be rapidly stored at -80±5 ° C or in liquid nitrogen, to determine the various complexes either in other day or distributed in two or more days after sample preparation (preferably consecutive days). It is recommended to divide the sample into aliquots to be thawed just before each enzymatic assay.

- **MITOCHONDRIA-ENRICHED PREPARATION**

1. Homogenization of fresh tissue or cell preparation should be performed immediately after sample collection, avoiding freezing steps, following previous reported protocols: Steps 1-9 of the 'Tissue Homogenization' protocol for fresh tissue mitochondria isolation and Steps 1 of the 'Cell lysate' protocol followed by Steps 4-9 from the 'Tissue Homogenization' protocol for fresh cells' mitochondria isolation. Steps 4-9 corresponding to the homogenization process should be ideally repeated by dissolving the resulting pellet in 1 ml of buffer to increase extraction efficiency. All along the mitochondrial isolation process, MB should contain 1 mg/ml of bovine serum albumin (BSA) to bring colloidal consistency to the buffer.

2. Fresh tissue homogenate or cell lysate supernatant (free of nucleus) should be afterwards centrifuged at 14,000 g at 4°C for 10 min to spin down organelles.

3. Discard the supernatant, wash the pellet with percoll 5% (v/v) and 1 mg/ml BSA in MB by posterior centrifugation (14,000 g at 4°C for 10 min) and dissolve the resultant pellet in 80 µl of MB containing 1 mg/ml of BSA.

4. Keep on ice at 0-6 ° C and calculate the total protein concentration subtracting protein content of MB (should be 1 mg/ml of BSA).

5. Dilute mitochondria enriched-suspension to 2 mg/ml. 100 µl is the recommended volume.

6. Respiratory chain complexes can be analysed within the same day in the mitochondria-enriched suspension. Alternatively, it should be rapidly stored at -80±5 ° C or in liquid nitrogen, to determine the various complexes either in other day or distributed in two or more days after sample preparation (preferably consecutive days). It is recommended to divide the sample into aliquots to be thawed just before the enzymatic assay.

(iii) ***Protein measurement:***

Total protein quantification can be performed according to the BCA protein assay (Pierce BCA Protein Assay Kit. Cat#23225), that combines the Biuret reaction with the colorimetric detection of the resulting cuprous ion (Cu^{1+}) by bicinchoninic acid (BCA).
The calibration curve (standards 1, 0.5, 0.25, 0.125, and 0.0625 mg protein/ml) is prepared with 2mg/ml BSA (Thermo Scientific, Cat#23210; liquid solution) and 0.15 M NaCl. These standards can be stored at -20 ° C (can be re-used 2-3 times).
Reagent is prepared by mixing the reagents A and B in a 50:1 ratio (mix 6.5 ml of Reagent A with 130 µL of reagent B, for the curve and three triplicate samples).
Dilute each sample to 1/4 and 1/8 in 0.15 M NaCl buffer. Add 10 µl of standards or diluted samples in each well. Then, add 200 µl of the reagent mixture A/B previously prepared. Incubate 30 minutes at 37°C and let cool down to room temperature for 5 minutes. Read the absorbance at a wavelength of 562 nm.
Proteins must be adjusted to 2mg/ml with MB before assaying MRC activities.

(vi) ***MRC enzyme assessment:***

Multicuvette carousel spectrophotometer makes possible the parallel read of 6 cuvettes simultaneously.
If the enzyme activity is very fast or not lineal (R factor below 0.975), it is recommended to dilute the sample with MB and repeat the assay.

- **COMPLEX I (NADH:ubiquinone oxidoreductase, CI)**

Procedure (for two patients and the control sample run in parallel):

1. Configure the spectrophotometer for 6 cuvettes, at 340 nm, 37°C and adjust to zero in the air.
2. Prepare 6 x 1-ml cuvettes (to analyse 3 with/3 without rotenone). Label with respective sample IDs, mark '+R' for rotenone inhibited samples.
3. Prepare the reaction medium in a 10 ml tube. Prepare always enough volume for an extra sample, i.e. for 7 cuvettes.

Reaction medium	Quantity/cuvette	Total quantity (n=7)
500 mM KP pH 7.5	100 µL	700 µL
25 mM Decylubiquinone	4 µL	28 µL
50 mg/ml BSA	75 µL	525 µL
Distilled water	746 µL	5222 µL
TOTAL	925 µL	6475 µL

4. Label three 5-ml tubes with respective sample Ids, add 2035µL of the reaction medium in each and 44µL of the respective muscle homogenate (diluted to 2 mg protein/ml).
5. Mix by gentle vortexing.
6. Transfer 945 µL to each cuvette.
7. Add 5 µL 2.5 mM rotenone to each '+R'-labelled cuvette, and 5 µL of a 1:1 Ethanol: DMSO mixture to the other cuvettes. CRITICAL STEP: to prevent precipitation of the rotenone and infra inhibition of the reaction, add 5 µL rotenone into the reaction mix, avoid rotenone solution to stick to the cuvette surfaces. CAUTION: protect rotenone from light. CAUTION: rotenone is toxic, avoid contact.
8. Mix by gentle vortexing.
9. Incubate the cuvettes at 37°C for 5 minutes in the spectrophotometer. CRITICAL STEP: read cuvettes in parallel during the run (with/without rotenone) for precise estimation of the inhibition.
10. Trigger the reaction by adding 50 µL NADH 2 mM (kept at room temperature; CAUTION: protect from light)
11. Mix the cuvettes by gentle vortexing.
12. Follow the decrease of absorbance during 3 minutes.
13. Calculate ΔAbs/min with and without rotenone and considering the reaction from 1 to 3 minutes.

The extinction coefficient for NADH is ε= 6.2 $mM^{-1}cm^{-1}$
The calculation factor is 4032.3 for 40 µg protein of muscle homogenate.
The specific activity of complex I is expressed as $nmols.minute^{-1}.milligram\ of\ protein^{-1}$, and is the result of subtracting the unspecific, non complex I activity measured with rotenone, from the total NADH:ubiquinone oxidoreductase activity.
Complex I activity = total activity without rotenone – unspecific activity with rotenone
Calculate the ratio Complex I activity / Citrate synthase activity.

- In case of cell suspension (fibroblasts or PBMC) CI activity is difficult to measure due to extensive unspecific NADH-dehydrogenase function and is strongly recommended treatment with percoll and digitonin following Chretien et al. 2003 methods (*Biochem Biophys Res Commun*. **301,** 222–224) [22].

- In case of mitochondria-enriched fraction, use the sample volume corresponding to 4 µg of protein and the calculation factor 40323.

- **COMPLEX II (succinate:ubiquinone oxidoreductase, CII)**

Procedure (for two patients and the control sample run in parallel):

1. Configure the spectrophotometer for 3 cuvettes, at 600 nm, 37°C and adjust to zero in the air.
2. Prepare 3 x 1-ml cuvettes. Label with respective sample identifications.
3. Prepare the reaction medium in a 10 ml tube. In all cases, prepare enough volume for an extra sample, i.e. for 4 cuvettes.

Reaction medium	Quantity/cuvette	Total quantity (n=4)
500 mM KP pH 7.5	50 µL	200 µL
200 mM Succinate	100 µL	400 µL
5mM DCPIP	10 µL	40 µL
10 mM KCN	100 µL	400 µL
50 mg/ml BSA	40 µL	160 µL
Distilled water	676 µL	2704 µL
TOTAL	976 µL	3904 µL

4. Add 976 µL of the reaction medium to each cuvette and 20 µL of the respective muscle homogenate (diluted to 2 mg protein/ml).
5. Mix by gentle vortexing.
6. Incubate the cuvettes at 37°C for 5 minutes, in the spectrophotometer.
7. Read baseline during 3 minutes.
8. Trigger the reaction by adding 4 µL of 25 mM decylubiquinone kept at room temperature.
9. Mix gentle by vortexing.
10. Follow the decrease of absorbance during 3 minutes.
11. Calculate ΔAbs/min considering the reaction from 0 to 3 minutes.

The extinction coefficient for DCPIP is ε=19.2 $mM^{-1}cm^{-1}$
The calculation factor is 1302.1 for 40 µg protein of muscle homogenate.
The specific activity of complex II is expressed as $nmol.min^{-1}.mg\ protein^{-1}$ and it is the result of subtracting the baseline (without decylubiquinone) from the total succinate:ubiquinone oxidoreductase activity
Complex II activity= total activity with decylubiquinone – baseline without decylubiquinone
Calculate the ratio Complex II activity / Citrate synthase activity.

- In case of cell suspension (fibroblasts or PBMC) use identical sample volume and calculation factor than for muscle homogenate.
- In case of mitochondria-enriched fraction, use the sample volume corresponding to 4 µg of protein and the calculation factor 13021.

- **COMPLEX I + III (NADH:cytochrome c oxidoreductase, CI+III)**

Procedure (for two patients and the control sample run in parallel):

1. Configure the spectrophotometer for 6 cuvettes, at 550 nm, 37°C and adjust to zero in the air.
2. Prepare 6 x 1-ml cuvettes (to analyse 3 with/3 without rotenone). Label with respective sample IDs, mark '+R' for rotenone inhibited samples.
3. Prepare the reaction medium in a 10 ml tube. In all cases, prepare enough volume for an extra sample i.e. for 7 cuvettes.

Reaction medium	Quantity/cuvette	Total quantity (n=7)
500 mM KP pH 7.5	100 µL	700 µL
50 mg/ml BSA	20µL	140 µL
10 mM KCN	100 µL	700 µL
1 mM cytochrome C	100 µL	700 µL
Distilled water	555 µL	3885 µL
TOTAL	875 µL	6125 µL

4. Label three 5-ml tubes with respective sample identifications, add 1936 µL of the reaction medium and 44 µL of the respective muscle homogenate (diluted to 2 mg/ml protein).
5. Mix by gentle vortexing.
6. Transfer 900 µL to each cuvette.
7. Add 5 µL 2.5 mM rotenone to each '+R'-labelled cuvette, and 5 µL of a 1:1 mixture of EtOH and DMSO to the other. CRITICAL STEP: to prevent precipitation of the rotenone and suboptimal inhibition of the reaction, drop the 5 µL rotenone into the reaction mix and not to the walls of the cuvettes. CAUTION: protect rotenone from light. CAUTION: rotenone is toxic, avoid contact.
8. Mix by gentle vortexing.
9. Incubate the cuvettes at 37°C for 5 minutes in the spectrophotometer. CRITICAL STEP: read cuvettes in parallel during the run (with/without rotenone) for precise estimation of the inhibition.
10. Trigger the reaction by adding 100 µL 2 mM NADH (kept at room temperature; CAUTION protect from light)
11. Mix the cuvettes by gentle vortexing.
12. Follow the decrease of absorbance during 3 minutes.
13. Calculate ΔAbs/min with and without rotenone and considering the reaction from 0 to 1.5 minutes

The extinction coefficient for cytochrome c is $\varepsilon = 18.5\ mM^{-1}cm^{-1}$

The calculation factor is 1351.4 for 40 µg protein of muscle homogenate

The specific activity of complex I+III is expressed in $nmol.minute^{-1}.milligram\ of\ protein^{-1}$, and is the result of subtracting the unspecific activity measured with rotenone, from the total NADH:cytochrome c oxidoreductase activity.

Complex I+III activity = total activity without rotenone – unspecific activity with rotenone.

Calculate the ratio Complex I+III activity / Citrate synthase activity.

- In case of cell suspension (fibroblasts or PBMC) use identical sample volume and calculation factor than for muscle homogenate or, if necessary, dilute 1/3 to 13.3 µg of protein and use 4054.2 as calculation factor.
- In case of mitochondria-enriched fraction, use the sample volume corresponding to 4 µg of protein and the calculation factor 13514.

- **COMPLEX II+III (succinate:ubiquinone-ubiquinol:cytochrome c oxidoreductase, CII+III)**

Procedure (for two patients and the control sample run in parallel):

1. Configure the spectrophotometer for four cuvettes, at 550 nm, 37°C and adjust to zero in the air.
2. Prepare 4 x 1-ml cuvettes. Label with respective sample identifications. The fourth cuvette is a control without sample.
3. Prepare the reaction medium in a 10 ml tube. Prepare always enough volume for an extra sample, i.e. for 5 cuvettes.

Reaction medium	Quantity/cuvette	Total quantity (n=5)
500 mM KP pH 7.5	40 µL	200 µL
50 mg/ml BSA	40 µL	200 µL
200 mM succinate	100 µL	500 µL
10 mM KCN	100 µL	500 µL
Distilled water	600 µL	3000 µL
TOTAL	880 µL	4400 µL

4. Add 880 µL of the reaction medium to each cuvette and 20 µL of the corresponding muscle homogenate (diluted to 2 mg protein /ml).
5. Mix by gentle vortexing.

OF NOTE: Prepare one more cuvette without adding the sample homogenate (with 20µL mannitol instead), to calculate the residual slope generated by the reagents (subtract that residual activity to the total enzymatic activity).

6. Incubate the cuvettes at 37°C during 5 minutes in the spectrophotometer.
7. Trigger the reaction by adding 100 µL of 1 mM cytochrome C (kept at room temperature).
8. Mix the cuvettes by gentle vortexing.
9. Follow the increase of absorbance during 3 minutes.
10. Calculate ΔAbs/min considering the reaction from 0 to 3 minutes.

The extinction coefficient of the cytochrome C is ε=18.5 $mM^{-1}cm^{-1}$

The calculation factor is 1351.4 for 40 µg protein of muscle homogenate.

The specific CII+III enzymatic activity is calculated as $nmol.min^{-1}.mg\ protein^{-1}$, and is the result of subtracting the residual activity without the sample, from the total succinate:ubiquinone-ubiquinol:cytochrome c oxidoreductase activity.

Calculate the ratio Complex II+III activity / Citrate synthase activity.

- In case of cell suspension (fibroblasts or PBMC) use identical sample volume and calculation factor than for muscle homogenate.
- In case of mitochondria-enriched fraction, use the sample volume corresponding to 4 µg of protein and the calculation factor 13514.

- **COMPLEX III (decylubiquinol: cytochrome C oxidoreductase, CIII)**

Procedure (for two patients and one control sample run in parallel):

1. Configure the spectrophotometer for 6 cuvettes, at 550 nm, 37 °C and adjust to zero in the air.
2. Prepare 6 x 1-ml cuvettes (to analyse 3 with/3 without antimycin A). Label with respective sample identifications, mark '+A' for antimycin A-inhibited samples.
3. Prepare the reaction medium in a 10 ml tube. Prepare always enough volume for an extra sample, i.e. for 7 cuvettes.

Reaction medium	Quantity/cuvette	Total quantity (n=7)
500 mM Phosphate buffer pH 7.5	50 µL	350 µL
1 mM Cytocrhome C	75 µL	525 µL
10 mM KCN	50 µL	350 µL
50 mM EDTA	2 µL	14 µL
Tween 20 2.5% (v/v)	10 µL	70 µL
Distilled water	789 µL	5523 µL
TOTAL	976 µL	6832 µL

4. Take 22 µL of each 2 mg protein/ml sample and dilute to 66 µL with homogenization buffer (3-fold dilution).
5. Label three 5-ml tubes with respective sample identifications, add 2147 µl of the reaction medium in each, and 22 µL of the respective diluted muscle homogenate from step 4.
6. Mix by gentle vortexing.
7. Transfer 986 µL to each cuvette (corresponding to three-fold diluted 20 µg of protein).
8. Add 4 µL of 2.5 mg/ml antimycin A solution to each '+A'-labelled cuvettes, and 4 µl of ethanol to the other cuvettes. CAUTION: Antimycin A is toxic; avoid contact.
9. Mix by gentle vortexing and incubate at 37°C for 5 min in the spectrophotometer. CRITICAL STEP: read cuvettes in parallel during the run (with/without antimycin A) for precise estimation of the inhibition.
10. Trigger CIII activity by adding 10 µl of 10 mM decylubiquinol solution. CAUTION: keep solution on ice and protect from light.
10. Mix the cuvettes by gentle vortexing.
11. Follow the increase of absorbance for 3 minutes.
12. Calculate ΔAbs/min with and without antimycin A and consider the reaction from 0 to 1.5 minutes.

The extinction coefficient for cytochrome C is ε= 18.5 $mM^{-1}cm^{-1}$

The calculation factor is 8108.1 for 3 fold-diluted 20 µg of protein.
The specific activity of complex III is expressed as nmol·min^{-1}·mg protein^{-1}, and is the result of subtracting the antimycin A-resistant from the total decylubiquinol: cytochrome c oxidoreductase activity.
Complex III activity = total activity without antimycin A– unspecific activity with antimycin A.
Calculate the ratio Complex III activity / Citrate synthase activity.

- In case of cell suspension (fibroblasts or PBMC) use identical sample volume and calculation factor as for muscle homogenate.
- In case of mitochondria-enriched fraction, use the sample volume corresponding to 3 fold-diluted 1 µg of protein and the calculation factor 162162.

- **COMPLEX IV (Cytochrome C oxidase, CIV)**

Procedure (for two patients and the control sample run in parallel):

1. Configure the spectrophotometer for three cuvettes, at 550 nm, 37 °C and adjust to zero in the air.
2. Prepare 3 x 1-ml cuvettes. Label with respective sample identifications.
3. Prepare the reduced cytochome c solution as described. Prepare always enough solution for an extra sample, i.e. for 4 cuvettes.

Reaction medium	Quantity/cuvette	Total quantity (n=4)
0.1 mM reduced cytochrome c	980 µL	3920µL

(The reaction medium should be ½ diluted in water in case of using fibroblasts)

4. Take 22 µL of each 2 mg protein/ml sample and dilute to 66 µL with homogenization buffer (3-fold sample dilution).
5. Add 980 µL of the reaction medium (1/2 diluted when using fibroblasts) to each cuvette and incubate at 37°C for 5 minutes in the spectrophotometer.
6. Trigger the reaction by adding 20 µL of the respective diluted muscle homogenate from step 4 (corresponding to three-fold diluted 40 µg of protein)
7. Mix the cuvettes by gentle vortexing.
8. Follow decrease of absorbance for 3 minutes.
9. Calculate ΔAbs/min and consider the reaction from 0 to 3 minutes.

The extinction coefficient for cytochrome C is ε= 18.5 mM^{-1}cm^{-1}
The calculation factor is 4054.2 for three-fold diluted 40 µg of protein.
The specific activity of complex IV is expressed in nmol·min^{-1}·mg protein^{-1}
Calculate the ratio Complex IV activity / Citrate synthase activity.

- In case of cell suspension (fibroblasts or PBMC) use identical sample volume and calculation factor as for muscle homogenate.
- In case of fibroblasts, the reaction medium should be ½ diluted.
- In case of mitochondria-enriched fraction, use the sample volume corresponding to three-fold diluted 1 µg of protein and the calculation factor 162168.

(v) *Citrate Synthase (CS) enzyme quantification*

Procedure (for two patients and the control sample run in parallel):

1. Configure the spectrophotometer for three cuvettes at 412 nm, 37 °C and adjust to zero in the air.
2. Prepare 3 x 1-ml cuvettes. Label with respective sample identifications.
3. Prepare the reaction medium in a 10 ml tube. Prepare always enough volume for an extra sample i.e. for 4 cuvettes.

Reaction medium	Quantity/cuvette	Total quantity (n = 4)
5 mM DTNB	20 µL	80 µL
10 mM acetyl-CoA	30 µL	120 µL
10% Triton X-100	10 µL	40 µL
1 M Tris-HCl pH 8.1	100 µL	400 µL
Distilled water	770 µL	3080 µL
TOTAL	930 µL	3720 µL

4. Transfer 930 µL of reaction medium in each cuvette
5. Add to each cuvette 20 µL of the respective sample muscle homogenate (diluted to 2 mg protein /ml).
6. Mix by gentle vortexing
7. Incubate the cuvettes at 37°C for 5 minutes.
9. Read the baseline for 4 minutes.
10. Trigger the reaction by adding 50 µL 10 mM oxaloacetate
11. Mix by gentle vortexing.
12. Follow the increase of absorbance during 4 minutes.
13. Calculate ΔAbs/min with and without oxaloacetate and analyse considering the reaction from 0 to 3 minutes.

The extinction coefficient for DTNB is ε= 13.6 $mM^{-1}cm^{-1}$
The calculation factor is 1838.2 for 40 µg protein of muscle homogenate.
The specific activity of citrate synthase is expressed in $nmol.min^{-1}.mg\ protein^{-1}$, and is the result of subtracting the unspecific activity (measured as baseline) from the total citrate synthase activity.
Citrate synthase = total activity– unspecific activity (baseline).

- In case of cell suspension (fibroblasts or PBMC) use identical sample volume and calculation factor as for muscle homogenate.
- In case of mitochondria- enriched fraction, use the sample volume corresponding to 1 µg of protein and the calculation factor 73528.
