# Supplementary material for: Multicentric Standardization of Protocols for the Diagnosis of Human Mitochondrial Respiratory Chain Defects
Source: Antioxidants (Basel). 2022 Apr 8;11(4):741. doi: 10.3390/antiox11040741 (PMC9027926; doi:10.3390/antiox11040741)
Supplement: Supplementary file 1 [file antioxidants-11-00741-s001.zip › antioxidants-1603519-Table S1.pdf]

Supplementary Table S1. Characteristics of the samples and individuals from the different sites of the study included as reference ranges (Table 3 and 5).

	**TABLE 3**										**TABLE 5**			
	**IBC U737[1] (n=119)**		**HCL U722[2] (n=29)**		**12O U723[3] (n=25)**		**VH U701[4] (n=14)**		**UPO U729[5] (n=75)**		**IBC U737[1] (n=28)**		**HCL U722[2] (n=11)**	
	Range	Mean ± SD	Range	Mean ± SD	Range	Mean ± SD	Range	Mean ± SD	Range	Mean ± SD	Range	Mean ± SD	Range	Mean ± SD
Age	0-75	41.5 ± 16.85	19 - 88	62.04 ± 17.90	0.02 - 70	26.16 ± 22.56	0.02 - 69	19.45 ± 22.72	0 - 95	23.47 ± 23.09	0,02-80	35.6± 17.85	27 - 86	65.0 ± 14.32
	Female/Male	% Female	Female/Male	% Female	Female/Male	% Female	Female/Male	% Female	Female/Male	% Female	Female/Male	% Female	Female/Male	% Female
Gender	65/54	54.6	17/12	58.6	10/15	40	5/9	30	NA	NA	17/11	30	6/5	54.5
Tissue	quadriceps		deltoids		quadriceps/biceps		quadriceps		NA		fibroblasts		PBMC	
Fasting	NO		NO		NO		YES		NO		NO		NO	

[1] Inborn Errors of Metabolism Section (IBC U737), Molecular Genetics and Biochemistry Service, Hospital Clínic of Barcelona, IDIBAPS and CSIC, Barcelona, Spain

[2] Muscle Research and Mitochondrial Function Laboratory (U722), Cellex-IDIBAPS, Faculty of Medicine and Health Sciences-University of Barcelona, Internal Medicine Department-Hospital Clínic of Barcelona, Spain

[3] Mitochondrial Diseases Laboratory (U723), Research Institute, Universitary Hospital 12 de Octubre (imas12), Madrid, Spain

[4] Biochemistry Service (U701), Universitary Hospital Vall d'Hebron, Barcelona, Spain

[5] Bioenergetics and Cell Physiology Service (U729), Central Services of Research, University Pablo de Olavide, Sevilla, Spain
