# Supplementary material for: Multicentric Standardization of Protocols for the Diagnosis of Human Mitochondrial Respiratory Chain Defects
Source: Antioxidants (Basel). 2022 Apr 8;11(4):741. doi: 10.3390/antiox11040741 (PMC9027926; doi:10.3390/antiox11040741)
Supplement: Supplementary file 1 [file antioxidants-11-00741-s001.zip › antioxidants-1603519-Table S2.pdf]

Supplementary Table S2. Representative data of the control porcine sample analyzed in the participating institutions, used both as an internal control (for daily intra-lab analyses) and external reference (inter-lab compaSrison).

	Complex	IBC U737 [1]	HCL U722 [2]	VH U701 [3]	12O U723 [4]	UPO U729 [5]	Mean	SD	CV
Specific	**CI**	43.50	41.42	44.10	44.60	45.76	43.85	1.60	3.66
	**CII**	43.80	38.28	33.00	99.50	40.73	46.79	27.36	58.49
	**CIII**	83.30	54.43	51.60	127.00	36.40	64.09	35.83	55.90
	**CI+CIII**	10.30	13.62	19.80	37.60	16.55	17.68	10.67	60.36
	**CII+CIII**	22.00	21.50	15.40	51.60	20.00	23.73	14.49	61.08
	**CIV**	76.10	75.97	77.80	266.80	67.71	95.93	86.14	89.79
	**CS**	112.30	136.64	153.20	291.20	146.02	158.48	70.65	44.58
Relative to Citrate Synthase (CS)	**CI / CS**	0.39	0.30	0.29	0.15	0.31	0.28	0.09	30.73
	**CII / CS**	0.39	0.28	0.22	0.34	0.28	0.30	0.07	22.61
	**CIII / CS**	0.74	0.40	0.34	0.44	0.25	0.40	0.19	46.18
	**CI+CIII / CS**	0.09	0.10	0.13	0.13	0.11	0.11	0.02	15.22
	**CII+CIII / CS**	0.20	0.16	0.10	0.18	0.14	0.15	0.04	24.67
	**CIV / CS**	0.68	0.56	0.51	0.92	0.46	0.61	0.18	30.02

[1] Inborn Errors of Metabolism Section (IBC U737), Molecular Genetics and Biochemistry Service, Hospital Clínic of Barcelona, IDIBAPS and CSIC, Barcelona, Spain
[2] Muscle Research and Mitochondrial Function Laboratory (U722), Cellex-IDIBAPS, Faculty of Medicine and Health Sciences-University of Barcelona, Internal Medicine Department-Hospital Clínic of Barcelona, Spain
[3] Mitochondrial Diseases Laboratory (U723), Research Institute, Universitary Hospital 12 de Octubre (imas12), Madrid, Spain
[4] Biochemistry Service (U701), Universitary Hospital Vall d'Hebron, Barcelona, Spain
[5] Bioenergetics and Cell Physiology Service (U729), Central Services of Research, University Pablo de Olavide, Sevilla, Spain
